# How does pole length affect lower back muscle activity at different inclines and skiing intensities during double poling?

**DOI:** 10.3389/fspor.2025.1438386

**Published:** 2025-02-18

**Authors:** Marie Lund Ohlsson, Marcus Nilsson, Mikael Swarén

**Affiliations:** ^1^ Department of Health Sciences, Swedish Winter Sports Research Centre, Mid Sweden University, Östersund, Sweden; ^2^ Department of Physiology, Nutrition and Biomechanics, The Swedish School of Sport and Health Sciences, Stockholm, Sweden; ^3^ Swedish Unit for Metrology in Sports, Department of Sports and Health Sciences, School of Health and Welfare, Dalarna University, Falun, Sweden

**Keywords:** cross-country skiing, back pain, EMG, injury, lumbar, sports

## Abstract

**Purpose:**

This study aimed to investigate how pole length, incline, and skiing intensity affect lower back muscle activation in elite cross-country skiers. This addressing a critical gap in understanding the biomechanical demands and risk of low back pain of double poling (DP).

**Method:**

Eleven elite cross-country skiers performed skiing trials on a treadmill, varying in incline (flat vs. 6°), intensity (two self-selected speeds, training speed and racing speed), and pole lengths. Muscle activity was measured by surface electromyography on the erector spinae thoracic and lumbar muscles, on the left and right side. A motion capture system was used for kinematic analysis of the lower back-pole moment arm and the hip angle during the DP cycle.

**Results:**

Compared to men, female skiers had a significantly higher (*p* < 0.001), overall muscle activation for the m. erector spinae lumbar on both the left and right side (26% of MVC vs. 15% of MVC, and 28% of MVC vs. 22% of MVC, for the left and right side, respectively). No correlations were found, neither between muscle activation to the lower back-pole moment arm, or to the hip angle.

**Conclusion:**

The findings suggest that female skiers experience significantly higher lumbar erector spinae activation during DP, potentially indicating greater susceptibility to back-related issues. The results also highlight the need to tailor training and right adapted equipment to mitigate lower back stress, especially in flat terrain high-intensity conditions. The asymmetrical muscle activity and gender differences underscores the need for further investigation into biomechanical factors influencing back muscle engagement in cross-country skiing.

## 1 Introduction

### 1.1 Epidemiology of back pain

Low back pain is a prevalent concern, affecting both the general population (1) and elite athletes (2). A systematic review by Trompeter et al. (3) highlighted a particularly high incidence of back pain-related issues among cross-country skiers compared to other sports. Over recent years, this problem has garnered attention within professional cross-country skiing, with some athletes requiring surgical interventions to continue their careers.

A retrospective epidemiological study conducted by Eriksson et al. (4) underscores the persistence of back problems among cross-country skiers over several years. This issue affects both senior elite cross-country skiers and students in ski high schools (5, 6). A study by Alricsson and Werner (7) studied the location of back pain among high school cross-country skiers, revealing that 47% of the participants reported past or present complaints of back pain, with 94% of them attributing their discomfort to the lumbar spine. The prevalence of low back pain among national elite-level cross-country skiers was also examined by Bahr et al. (5), who found that 63% of the surveyed skiers had experienced low back pain in the preceding 12 months. This study (5) suggests that periods of intensified training and competition may contribute to an increased occurrence of low back pain in cross-country skiers.

### 1.2 Training and lumbar stress

Endurance athletes with high training volumes and repetitive back-loading activities, such as rowing and skiing, face elevated risk of and chronic lumbar issues (3, 8). In cross-country skiing, repetitive lumbar flexion and loading, particularly during double poling (DP), may contribute to this heightened risk (5). Cross-country skiing is a complex and physically demanding sport requiring years of systematic, low and high-intensity training to achieve elite performance. Elite skiers often accumulate 750–950 annual training hours, with a significant proportion of summer roller skiing (9, 10). This extensive training load, including prolonged DP-specific sessions and roller skiing, places significant biomechanical stress on the lumbar spine, which may be influenced by factors such as pole length and terrain. Interestingly, Alricsson and Werner (7) found no statistically significant differences between pre-season and in-season reports of symptoms or injuries among high school skiers.

In classic long-distance ski races, such as the 90 km Vasaloppet, most elite skiers rely exclusively on DP throughout the race (11). To sustain efficient technique and high speeds in long-distance races, skiers engage in extensive DP-specific training, incorporating prolonged sessions and supplemental upper-body core and strength training (12).

### 1.3 Technique and biomechanical demands

In addition to training load, skiing technique is a critical factor in cross-country skiing performance and injury prevention. In recent decades, DP has evolved into a more dynamic and biomechanically complex technique, with greater lower-body engagement to enhance efficiency and speed (12–14). Efficient DP technique is characterized by increased joint flexion, higher flexion velocities, and greater pole force during shorter poling phases (15). This evolvement of DP has resulted in a more explosive sub-technique, capable of generating high speeds, while exposing the body to greater peak forces (14).

Exclusive use of DP requires skiers to adapt their technique to different terrains. Biomechanical differences between flat and uphill DP are particularly relevant to understanding how terrain affect lumbar muscle activation and technique (14). In uphill DP, compared to flat terrain, cycle length decreases, frequency increases, and peak pole force is both larger and occurs later in the poling phase (14). For flat terrain, Jonsson et al. (16) found that faster skiers exhibit a greater forward body lean and more vertical pole placement at pole plant compared to slower skiers.

Holmberg et al. (15) analysed muscle activation sequencing during the DP cycle, reporting low m. erector spinae activation at pole plant and recovery, with moderate activation at the end of the poling phase. These findings provide a foundation for understanding lumbar muscle activity in DP and its potential relationship to pole length and terrain. Subsequent studies (17, 18) have demonstrated increased m. erector spinae activity with higher skiing speeds. Prior research has primarily used unilateral electrode placement as DP is a symmetrical motion; however, from an EMG perspective, the muscle activity may appear otherwise. Notably, Renkawitz et al. (19) identified a significant link between neuromuscular imbalance in the erector spinae and low back pain among elite amateur tennis players. Similarly, Mazis (20) reported increased and asymmetric erector spinae activity in individuals with back pain. These findings underscore the importance of bilateral analysis of m. erector spinae activity during DP to identify potential asymmetries that may contribute to lumbar stress or low back pain in cross-country skiers.

#### 1.3.1 Pole length

Carlsen et al. (21) found that longer poles (up to 10 cm above self-selected length) reduced O2-cost during DP, particularly on moderate inclines compared to flat terrain. Additionally, longer poles were associated with extended ankle, knee, and hip joint angles, resulting in a more upright posture among participants (21). A separate study by Onasch et al. (22) examined pole lengths ranging from 77% to 98% of participants’ body height and found that longer poles enhanced poling efficiency and propulsive impulse while reducing metabolic cost during DP. Improved skiing efficiency with longer poles may mitigate muscular fatigue and support better maintenance of DP technique, potentially reducing the risk of back pain. However, further research is needed to confirm these benefits. In addition, these findings suggest that longer poles may reduce lumbar stress by promoting a more upright posture, which could influence activation patterns in the lumbar back muscles. However, the specific relationship between pole length and lumbar muscle activation during varying inclines and intensities remains unclear and warrants further investigation.

Remaining injury-free is essential for elite athletes to sustain top-level performance over multiple years (23). Understanding injury prevention, muscle activation, and movement patterns is therefore crucial for advancing elite athletes' training. Although research has advanced our understanding of DP mechanics, the specific effects of incline, intensity, and pole length on lumbar muscle activation remain poorly understood. Given the increasing use of DP in all type of terrain (11, 24, 25) this gap in knowledge is critical to address. Gaining this knowledge could enhance training programs and preventive strategies, providing valuable insights for coaches, athletes, medical teams, and researchers. Hence, this study aims to investigate how lumbar muscle activation is influenced by incline, intensity, and pole length during roller-skiing on a treadmill, comparing flat vs. uphill terrain and high vs. low intensity.

## 2 Method

Eleven elite, professional cross-country skiers (seven males and four females; age: 25 ± 1 years, height: 177 ± 6 cm, weight: 71 ± 9 kg) who competed at national and international elite level volunteered to participate in this study. Five of the participants had suffered from lumbar pack pain, previously. All participants were healthy and uninjured at the time of data collection, and no one had any anatomical anomalies. All tests were conducted on a motorised treadmill specifically designed for roller skiing (Rodby Innovation AB, Vänge, Sweden). All participants used standardised roller skis (Swix Roadline Classic with Rottefella bindings). The participants wore their own ski boots and were assigned poles of different lengths. Pole baskets were exchanged for baskets designed for treadmill roller-skiing.

The research study and experimental protocol were pre-approved by the Swedish Ethical Review Authority (Dnr 2021-06327-01). All research was conducted in accordance with the Code of Ethics of the World Medical Association (Declaration of Helsinki).

### 2.1 Test protocol

**Figure 1:**
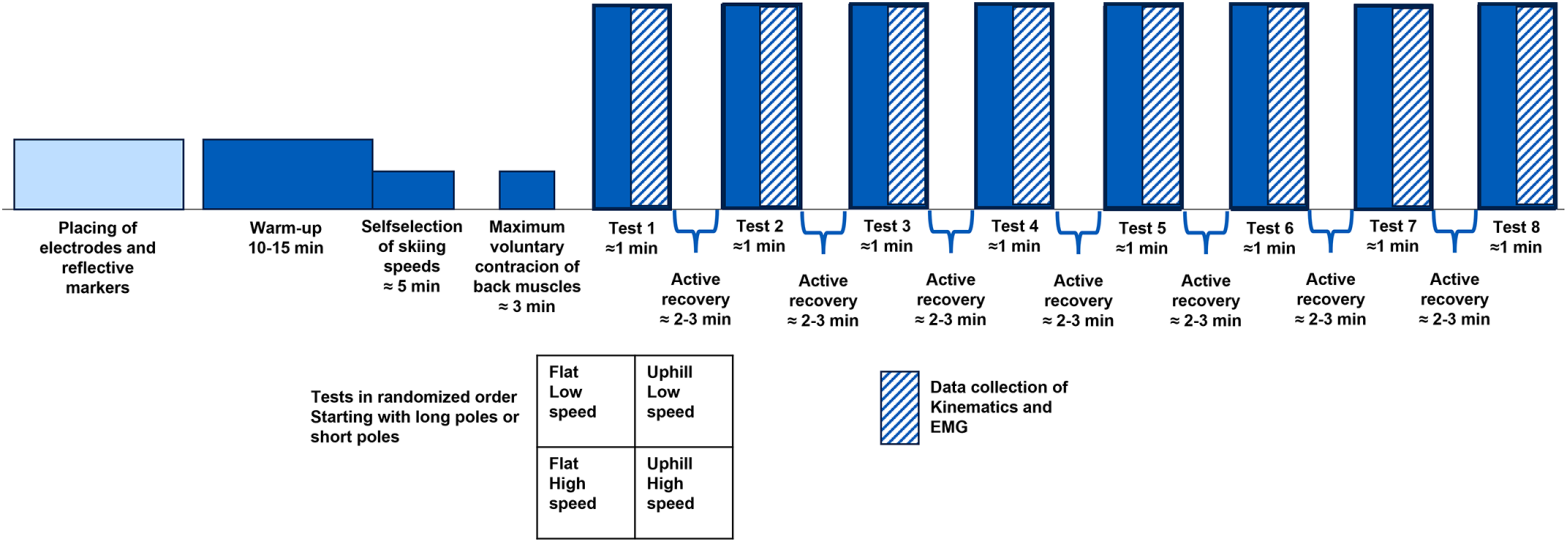
A schematic visualisation of the test protocol.

The test protocol consisted of eight double-poling (DP) skiing subsets performed: on a flat surface and on an uphill surface, with an inclination of six degrees, at two different speeds and with two different pole lengths. The different skiing speeds were self-selected and defined as a long-distance training speed and a self-selected race speed, mimicking the speed during a 10 km and a 15 km race with induvial start, for females and males, respectively. The different pole lengths where the participants’ personal classic poles (maximal 83% of body height) and skate poles (approximately 90% of body height). The order of the subsets was randomly selected for each participant to minimize potential sequence effects. A schematic experimental design is presented in Figure 1.

The participants performed a 10–15 min individual warm-up on the treadmill with self-selected speed and inclination. Following the warm-up, the participants familiarised with the different subsets as well as choosing the self-selected skiing speeds. Each subset started with DP for 30 s before collecting 3D kinematics and EMG data. Data were collected for approximately 30 s to include ten continuous poling cycles in the analysis. After data collection, the treadmill speed was reduced to a self-selected speed, enabling active recovery for approximately 2–3 min. If the next subset required a different pole length, the poles were changed while the participant skied slowly (diagonal stride, legs only) on the treadmill during the recovery phase.

### 2.2 Muscle activity (EMG)

Muscle activity was measured using surface electromyography (EMG), Ultium EMG (Noraxon USA Inc., USA) at a sampling frequency of 2000Hz. The participants were equipped with wireless EMG electrodes on the skin to measure the muscle activation during DP in the low back muscles, m. erector spinae thoracic and lumbar, on the left and right side. The electrodes (Ambu BlueSensor, Ambu A/S, Ballerup, Denmark) were self-adhesive and applied directly to the skin. Prior to electrode placement, body hair was shaved off, and the skin was disinfected with alcohol before placing the electrodes. The electrodes were placed parallel to the muscle fibre direction with an inter-electrode distance of 2 cm. To normalize EMG muscle activation data, participants executed a maximum voluntary contraction (MVC) for the back muscles following the protocol by Ball and Scurr (26). This involved a spine extension against static resistance while lying prone. Each MVC lasted 2–3 s and was executed twice, with a 30 s rest period between trials. The first trial served for familiarization, while the second trial was used to establish the MVC reference values. EMG signals recorded during the second trial provided the 100% MVC reference for all subsequent subsets in the study and to compare the activation level between different muscles. Continuous muscle activity was analysed for all muscles and is presented as the average activation during the ten poling cycles. A band-passed filter (10–300 Hz) was used for the EMG signal to remove low and high-frequency noise and a root mean square algorithm (50 ms) was used for smoothing.

### 2.3 Kinematic analysis

To analyse the -lower back-pole moment arm and the hip angle during the DP cycle, each athlete was equipped with reflective markers directly on the skin. Each marker had a diameter of 12.5 mm and was attached by double-sided tape and additionally fixed with tape around the base to avoid movement of the markers. Markers were strategically placed at key anatomical landmarks, including left and right ASIS (Anterior Superior Iliac Spine), the left and right PSIS (Posterior Superior Iliac Spine), the left and right greater trochanter, the left and right acromion, medial and lateral knee joint on the left and right leg, and on the top and bottom of the poles. Kinematic data were collected by nine Qualisys Miqus M3 cameras (Qualisys AB, Gothenburg, Sweden) at 200 Hz. All cameras were mounted, high on the walls or on tripods around the treadmill to ensure comprehensive coverage of the measurement volume. The measurement volume was calibrated with a hand-held T-wand, consisting of two reflective markers at each end, with a known distance between them. The orientation of the coordinate system was performed by placing an L-frame at the decided origin which was in the lower right corner of the treadmill. Additionally, a 2D Video (60 fps) was used to record and identify DP cycles.

The lower back-pole moment arm was determined as the perpendicular distance between the pole and the PSIS marker. The point of perpendicular attachment on the pole shifts throughout the poling phase due to changes in the pole's inclination and, consequently, alterations in the direction of the ground reaction force vector, which is assumed to align with the direction of the pole. Given that DP is a symmetric motion and symmetry between left and right side was assumed. Hence, only the moment arm for the right side was calculated. Data analysis was conducted using Qualisys Track Manager 2019.2 (Qualisys AB, Gothenburg, Sweden), Matlab R2017a (The MathWorks Inc., Natick, MA, USA) and Microsoft Excel (Microsoft Corp. Inc., Redmond, WA, USA).

### 2.4 Statistical analysis

All data were checked for normality, using the Shapiro–Wilk test and neither the lower back-pole moment arm data or hip angle data conformed to a normal distribution (*p* < 0.05), whereas some of the EMG datasets were normally distributed (*p* > 0.05). Hence, a Kruskal–Wallis rank sum test was used for the moment arm data, with a Dwass-Steel-Critchlow-Flinger test for pairwise comparisons if there was a global significance for the Kruskal–Wallis test. The hip angle data were analysed using a Mann–Whitney U test and the EMG data sets were analysed with either a Wilcoxon signed-rank test or a paired *t*-test, depending on the normality of the data sets. A two-tailed *p* value of <0.05 was considered as statistically significant. All statistical analyses were performed in SPSS (IBM Inc., Armonk, NY, USA) and Jamovi statistical software (27). The kinematic data are presented as median [IQR], whereas the skiing speeds are presented as mean ± SD. For comparison reasons, EMG data are presented as mean ± SD in tables.

## 3 Results

For female skiers, the self-selected skiing speeds for flat vs. incline skiing were 20 ± 1 km/h and 23 ± 1 km/h, vs. 7 ± 0 km/h and 10 ± 1 km/h, for training and race speed, respectively. For male skiers, skiing speeds for flat vs. incline skiing were 23 ± 1 km/h and 28 vs. 1 km/h vs. 10 ± 1 km/h and 13 ± 2 km/h, for training and race speed, respectively. EGM data for one skier were removed due to poor electrode connections.

The asymmetry of m. erector spinae lumbar was significantly (<0.001) larger compared to m. erector spinae thoracic, where the overall median activation of m. erector spinae lumbar was significantly higher on the right side compared to the left side [24% [10%] of MVC vs., 19% [12%] of MVC, *p* < 0.001]. Compared to male skiers, female skiers had a significantly higher (*p* < 0.001), overall muscle activation for the m. erector spinae lumbar on both the left and right side (26% [9%] of MVC vs., 15% [9%] of MVC, and 28% [10%] of MVC vs. 22% [8] of MVC, for the left and right side, respectively). Total EMG results for different pole lengths, inclinations and skiing intensities are presented in Tables 1–3, respectively.

**Table 1:** Average EMG-activation (% of MVC) for short Poles compared to long Poles.

Erector spinae lumbar left				
	Short poles	Long poles	*p* (Wilcoxon)	*p* (*t*-test)
Low intensity—flat	19 ± 9	20 ± 8	–	0.372
Low intensity—uphill	20 ± 20	22 ± 20	0.953	–
High intensity—flat	23 ± 10	26 ± 9	–	0.109
High intensity—uphill	15 ± 7	19 ± 7	–	**0**.**035**
Erector spinae thoracic left				
	Short poles	Long poles	*p* (Wilcoxon)	*p* (*t*-test)
Low intensity—flat	17 ± 7	17 ± 4	–	0.977
Low intensity—uphill	19 ± 12	19 ± 10	0.767	–
High intensity—flat	18 ± 7	19 ± 4	–	0.868
High intensity—uphill	19 ± 10	19 ± 7	–	0.876
Erector spinae lumbar right				
	Short poles	Long poles	*p* (Wilcoxon)	*p* (*t*-test)
Low intensity—flat	26 ± 9	25 ± 9	–	0.41
Low intensity—uphill	27 ± 18	27 ± 20	0.678	–
High intensity—flat	29 ± 20	30 ± 8	–	0.614
High intensity—uphill	25 ± 8	25 ± 7	–	0.934
Erector spinae thoracic right				
	Short poles	Long poles	*p* (Wilcoxon)	*p* (*t*-test)
Low intensity—flat	17 ± 3	17 ± 5	–	0.635
Low intensity—uphill	20 ± 14	20 ± 12	0.767	–
High intensity—flat	18 ± 5	19 ± 6	–	0.145
High intensity—uphill	17 ± 5	19 ± 6	0.086	–

Wilcoxon signed-rank test (*p*-value), paired *t*-test (*p*-value). Bold *p*-value indicates significant difference.

**Table 2:** Average EMG-activation (% of MVC) for flat compared to uphill skiing.

Erector spinae lumbar left				
	Flat	Uphill	*p* (Wilcoxon)	*p* (*t*-test)
Low intensity—short pole	19 ± 9	20 ± 20	0.26	–
Low intensity—long pole	20 ± 8	22 ± 20	0.11	–
High intensity—short pole	23 ± 10	15 ± 7	–	**0.001**
High intensity—long pole	26 ± 9	19 ± 7	–	**0.001**
Erector spinae thoracic left				
	Flat	Uphill	*p* (Wilcoxon)	*p* (*t*-test)
Low intensity—short pole	17 ± 7	19 ± 12	–	0.593
Low intensity—long pole	17 ± 4	19 ± 10	0.953	–
High intensity—short pole	18 ± 7	19 ± 10	–	0.624
High intensity—long pole	19 ± 4	19 ± 7	–	0.996
Erector spinae lumbar right				
	Flat	Uphill	*p* (Wilcoxon)	*p* (*t*-test)
Low intensity—short pole	26 ± 9	27 ± 18	0.441	–
Low intensity—long pole	25 ± 9	27. ± 20	0.374	–
High intensity—short pole	29 ± 20	25 ± 8	–	0.186
High intensity—long pole	30 ± 8	25 ± 7	–	**0.005**
Erector spinae thoracic right				
	Flat	Uphill	*p* (Wilcoxon)	*p* (*t*-test)
Low intensity—short pole	17 ± 3	20 ± 14	0.953	–
Low intensity—long pole	17 ± 8	20 ± 12	0.859	–
High intensity—short pole	18 ± 5	17 ± 5	0.110	–
High intensity—long pole	19 ± 6	19 ± 6	–	0.955

Wilcoxon signed-rank test (*p*-value), paired *t*-test (*p*-value). Bold *p*-value indicates significant difference.

**Table 3:** Average EMG-activation (% of MVC) for low compared to high skiing intensity.

Erector spinae lumbar left				
	Low intensity	High intensity	*p* (Wilcoxon)	*p* (*t*-test)
Flat—short pole	19 ± 9	23 ± 10	–	**0.014**
Uphill—short pole	20 ± 20	15 ± 7	0.859	–
Uphill—long pole	22 ± 20	19 ± 7	0.214	–
Flat—long pole	20 ± 8	26 ± 9	–	**0.003**
Erector spinae thoracic left				
	Low intensity	High intensity	*p* (Wilcoxon)	*p* (*t*-test)
Flat short pole	17 ± 7	18 ± 7	–	**0.042**
Uphill short pole	19 ± 12	19 ± 10	–	0.782
Uphill long pole	19 ± 10	19 ± 7	0.139	–
Flat long pole	17 ± 4	19 ± 4	–	**0.002**
Erector spinae lumbar right				
	Low intensity	High intensity	*p* (Wilcoxon)	*p* (*t*-test)
Flat short	26 ± 9	29 ± 20	–	**0.009**
Uphill short	27 ± 18	25 ± 8	0.441	–
Uphill long	27 ± 20	25 ± 7	0.139	–
Flat long	25 ± 9	30 ± 8	–	**0.001**
Erector spinae thoracic right				
	Low intensity	High intensity	*p* (Wilcoxon)	*p* (*t*-test)
Flat short	17 ± 3	18 ± 5	–	0.127
Uphill short	20 ± 14	17 ± 5	0.441	–
Uphill long	20 ± 12	19 ± 6	0.110	–
Flat long	17 ± 5	19 ± 6	–	0.141

Wilcoxon signed-rank test (*p*-value), paired *t*-test (*p*-value). Bold *p*-value indicates significant difference.

**Figure 2:**
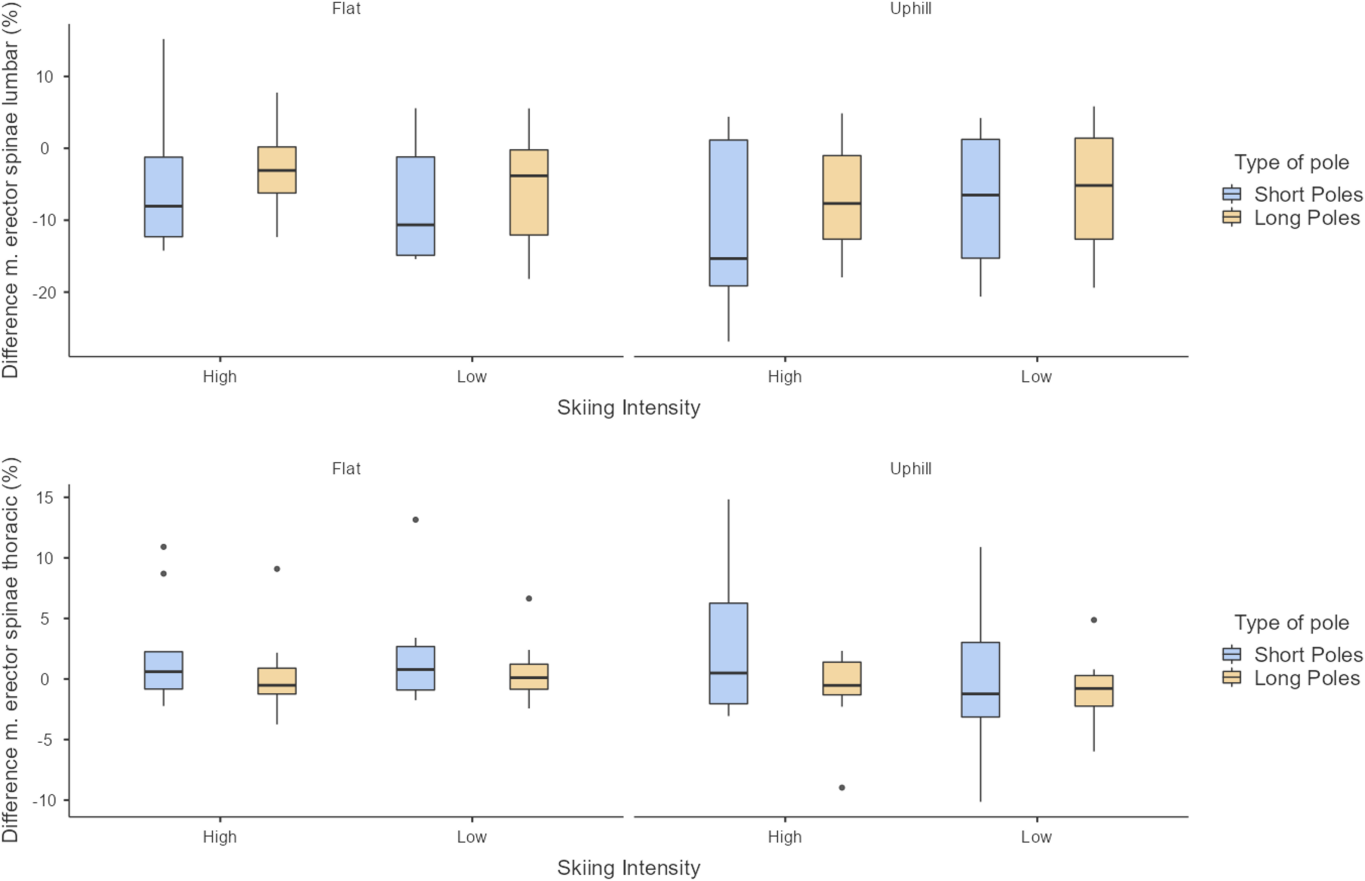
Box plots of the asymmetrical muscle activation between the left and right side, of the m. erector spinae lumbar (top) and the m. erector spinae thoracic (bottom). Positive value indicates a higher muscle activation on the left side.

Figure 2 shows the difference in muscle activation between the left and the right side for m. erector spinae lumbar and m. erector spinae thoracic. Identified outliers belong to three different skiers, where one skier has four outliers, all with classic poles, one skier has three, all during flat skiing and one skier has one outlier during uphill skiing with skate poles.

The median of the lower back-pole moment arm was significantly longer during skiing with classic ski poles compared to skating poles [39% [7%] of body height vs. 38% [9%] of body length, *p* < 0.05]. However, no correlations were found between the lower back—pole moment arm and muscle activation.

Female skiers exhibited significantly smaller median minimum and maximum hip angles compared to male skiers. The median minimum hip angle was 81° [8°] for females and 84° [11°] for males (*p* < 0.001), while the median maximum hip angle was 161° [17°] for females and 168° [14°] for males (*p* < 0.001). Both minimum and maximum hip angles were significantly affected by skiing speed, inclination and type of poles, see Table 4.

**Table 4:** Median and IQR for minimum and maximum hip angles.

Type of poles			
	Classic poles	Skate poles	*p* value
Maximum hip angle	167° (16°)	168° (16°)	**0.003**
Minimum hip angle	81° (12°)	85° (11°)	**<0.001**
Inclination			
	Flat	Uphill	*p* (*t*-test)
Maximum hip angle	172° (10°)	160° (12°)	**<0.001**
Minimum hip angle	81° (11°)	84° (11°)	**0.005**
Skiing speed			
	Low Intensity	High Intensity	*p* (*t*-test)
Maximum hip angle	168° (16°)	167° (15°)	0.182
Minimum hip angle	88° (10°)	78° (7°)	**<0.001**

Bold *p*-value indicates significant difference. A hip angle of 180° equals anatomic position.

Maximum hip angle was positively correlated to the lower back-pole moment arm [*ρ*(80) = 0.569, *p* < 0.001]. No correlations were found between the hip angles and muscle activation.

## 4 Discussion

The primary findings of this study are summarised as follows: (i) Effect of Pole Length on Muscle Activation: During uphill high-intensity DP, the use of long poles resulted in significantly greater muscle activation in the left m.erector spinae lumbar compared to short poles (*p* = 0.035, Table 1). (ii) Terrain Influence on Muscle Activation: High-intensity DP on flat terrain, compared to uphill DP with long poles, led to significantly higher activation in the left m.erector spinae lumbar (*p* < 0.001) and right m.erector spinae lumbar (*p* = 0.005). Similarly, high-intensity flat terrain DP compared to uphill DP with short poles produced higher activation in the left m.erector spinae lumbar (*p* = 0.001, Table 2). (iii) Intensity and Terrain Effects: High-intensity DP on flat terrain with short poles elicited significantly greater muscle activation than low-intensity DP in the left m.erector spinae lumbar (*p* = 0.014), right m.erector spinae lumbar (*p* = 0.01), and left m.erector spinae thoracic (*p* = 0.042). Additionally, during flat terrain DP with long poles, high-intensity DP induced greater activation compared to low-intensity DP in the left m.erector spinae lumbar (*p* = 0.003), left m.erector spinae thoracic (*p* = 0.002), and right m.erector spinae lumbar (*p* = 0.001, Table 3).

Uphill high-intensity DP with long ski poles, in comparison to employing short ski poles, resulted in a significantly elevated level of muscle activation in the left erector spinae lumbar muscle (*p* = 0.035, Table 1). (ii) When analysing high-intensity flat terrain DP as opposed to uphill DP with long poles, there was a notable increase in muscle activation observed in the following muscle groups: left erector spinae lumbar (*p* < 0.001) and right erector spinae lumbar (*p* = 0.005). Furthermore, high-intensity flat terrain DP, when contrasted with uphill DP using short poles, also produced a higher level of muscle activation in the left erector spinae lumbar muscle (*p* = 0.001) (Table 2). (iii) Comparing high-intensity DP to low-intensity DP, it was observed that flat terrain DP with short poles exhibited higher muscle activation in the left erector spinae lumbar muscle (*p* = 0.014), right erector spinae lumbar muscle (*p* = 0.01), and left erector spinae thoracic muscle (*p* = 0.042) (Table 3). Additionally, high-intensity compared to low intensity on flat terrain DP with long poles, a greater level of muscle activation was detected in the left erector spinae lumbar muscle (*p* = 0.003), left erector spinae thoracic muscle (*p* = 0.002), and right erector spinae lumbar muscle (*p* = 0.001), (Table 3).

The m. erector spinae lumbar exhibited greater asymmetry between the left and the right side compared to the m. erector spinae thoracic. As shown in Figure 2, the lumbar muscles also demonstrated a larger spread and variability in asymmetry, indicated by a broader interquartile range (IQR). Skiing with classic poles further increased this variability, particularly in the m. erector spinae thoracic during uphill skiing. This variability is likely influenced by differing DP strategies, where skiers either employ a high poling frequency with lower impulse per cycle or a lower frequency with higher impulse per cycle. Additionally, outliers in Figure 2 were attributed to three different skiers, primarily during flat skiing. Notably, two skiers accounted for seven of the total eight outliers. One skier consistently exhibited high asymmetrical activation of the m. erector spinae thoracic across all trials with classic poles, while the other displayed three outliers during flat skiing with both skating and classic poles. To understand the reasons behind these asymmetrical activation patterns, additional muscles should be monitored to provide a comprehensive view of the activation chain during the DP cycle. If EMG measurements can reliably identify instances of sustained asymmetrical muscle activation, incorporating such assessments into annual testing protocols for elite skiers could improve understanding of asymmetrical activation and its potential link to lumbar back pain.

On a group level, female skiers had higher muscle activity in the erector spinae lumbar muscles, compared to male skiers. However, the results from the present study cannot fully explain this difference. A plausible explanation might be the difference in hip angle, where female skiers had smaller hip angles, resulting in greater flexion with a larger forward lean of the upper body, and hence larger strain on the back muscles. In addition, the muscles on the left side showed larger variations in muscle activity between the different scenarios, compared to the muscles on the right side. Interestingly, Renkawitz et al. (19) observed a significant association between neuromuscular imbalance of erector spinae and the occurrence of low back pain among elite amateur tennis players. In addition, Mazis (20) investigated muscle activity of the erector spinae muscle group during functional movements, and found that there was an increased and asymmetric muscle activity of the erector spinae muscles among the participants with back pain. Hence, one can speculate that the differences in muscle activation between the left and right side in the present study might increase the risk of lower back pain. Still, every athlete has unique anatomical characteristics that may influence muscle activation patterns, even in symmetric movements, such as DP. For example, slight differences in leg length, spinal alignment, or muscle strength between the left and right sides could affect how muscles are recruited during DP. Identifying neuromuscular imbalances in healthy athletes might provide an opportunity for early intervention as addressed by Renkawitz et al. (19). Addressing these imbalances through targeted training or technique adjustments could help prevent future injuries, such as low back pain, even if the athletes are currently asymptomatic. Hence, the research about asymmetric muscle activity and back pain among cross-country needs further investigation.

The comparative analysis between flat and uphill DP conducted in this study revealed that in the context of lumbar spinae muscle activation, flat, high-intensity DP with long poles induced a more pronounced response when compared to uphill DP. A similar pattern was observed in flat, high-intensity DP with short poles, albeit limited to the left lumbar spinae. These observations suggest that the flat, high-intensity DP might exert greater stress on the lower back muscles compared to low-intensity skiing. Biomechanical disparities between flat and uphill DP have been investigated by Stöggl and Holmberg (14), who reported that uphill DP leads to shorter cycle lengths, higher frequencies, and greater peak pole forces occurring later in the poling phase (14). Presently, uphill DP is characterized by a “pumping” motion, which may imply increased workload on the leg muscles and reduced strain on the back muscles. The study by Stöggl and Holmberg (14) further demonstrated that uphill DP necessitates greater engagement of the lower body. The adaptability of DP techniques in response to varying terrains may account for the observed alterations in muscle activity during uphill DP.

Regarding the comparison of intensity levels, the present study observed heightened muscle activation in the back muscles at higher intensities, which is consistent with findings by Zoppirolli et al. (18). Previous studies (14, 28) have shown that the DP swing phase, decreases with increased skiing speed, resulting in a faster hip and back extension, which explains the increased muscle activity of the lower back at higher skiing intensities. The ability of athletes to achieve higher speeds has exposed their bodies to greater peak forces (14). This has implications for increased overall back loading during extended periods of intense DP training and competition. Additional research is essential to deepen our understanding of how repeated high-intensity DP affects the back health of skiers, longitudinally.

The current study only investigated DP, even though cross-country skiing consists of several different sub techniques. However, as mentioned by other studies (11, 12, 14, 24), DP has become the dominant sub technique in classic cross-country skiing, which is why only DP was investigated. Interestingly, research show that diagonal skiing style is the most commonly back pain-inducing style, among elite cross-country skiers, age 16–25 years (4, 29). A further limitation of this study is the small sample size (11 participants), which restricts the generalisability of the findings to the wider population of elite cross-country skiers. However, previous DP research involving elite and professional skiers has typically included a similar number of participants (11–13), reflecting the inherent challenges in accessing this specific athlete population (12, 14, 15, 28, 30). Additionally, the use of a motorised treadmill for roller skiing may not perfectly replicate the dynamics and biomechanical conditions of outdoor skiing, potentially affecting the ecological validity of the results. Moreover, while EMG measurements were taken bilaterally for the m. erector spinae, other muscles contributing to the double-poling motion were not assessed, which could provide a more comprehensive understanding of muscle coordination and activation. Hence, future research should include EMG measurement of additional muscles, during different sub-techniques while skiing on snow.

In conclusion, this study has shed light on the intricate relationship between terrain, intensity levels, and muscle activation patterns in the lower back during DP. High-intensity flat terrain DP induces notable increases in muscle activation in the erector spinae lumbar muscles compared to uphill DP, suggesting that high intensity skiing on flat terrain, may pose greater stress on the lower back muscles. The observation of asymmetrical muscle activity between the left and right sides, alongside the gender differences in muscle activation, underscores the need for further investigation into biomechanical factors influencing back muscle engagement in skiing. Additionally, exploring other sub-techniques of cross-country skiing will offer a comprehensive understanding of the sport's biomechanical demands and potential implications for athletes’ well-being.

## Data Availability

The datasets presented in this article are not readily available because participants did not consent to share the data outside of the research team. Requests to access the datasets should be directed to miv@du.se
